# Rickettsial Diseases: Not Uncommon Causes of Acute Febrile Illness in India

**DOI:** 10.3390/tropicalmed5020059

**Published:** 2020-04-15

**Authors:** Manisha Biswal, Sivanantham Krishnamoorthi, Kamlesh Bisht, Amit Sehgal, Jasleen Kaur, Navneet Sharma, Vikas Suri, Sunil Sethi

**Affiliations:** 1 Department of Medical Microbiology, Postgraduate Institute of Medical Education and Research, Chandigarh 160012, India; drsivananthamk@gmail.com (S.K.); kamalbisht120@gmail.com (K.B.); sehgal107@gmail.com (A.S.); jasleen84@yahoo.com (J.K.); sunilsethi10@hotmail.com (S.S.); 2 Department of Microbiology, All India Institute of Medical Sciences, Bathinda, Punjab 151001, India; 3 Department of Internal Medicine, Postgraduate Institute of Medical Education and Research, Chandigarh 160012, India; navneetsharma@hotmail.com (N.S.); surivikas9479@gmail.com (V.S.)

**Keywords:** rickettsioses, rickettsia species, SFG, TG, acute febrile illness, India, *glt*A PCR

## Abstract

Rickettsial diseases (RDs) are major under-diagnosed causes of arthropod borne acute febrile illness (AFI) presenting with a range of symptoms from mild self-limiting fever to fatal sepsis. The spotted fever group (SFG) and typhus group (TG) are major RDs, which are commonly caused by *Rickettsia conorii* and *Rickettsia typhi*, respectively. The limited availability and role of serological tests in the acute phase of illness warrants rapid reliable molecular methods for diagnosis and epidemiological studies. Two hundred patients with AFI in whom the routine fever diagnostics were negative, were enrolled over a period of two months (April 2019 to May 2019). DNA was extracted and in-house nested PCR using primers specific for both SPG and TG pathogens was used. The positive amplified products were sequenced for species identification and phylogenetic analysis was performed using MEGA 7.0.14 software (iGEM, Temple University, Philadelphia, PA 19122, USA). The demographic details of the RD cases were documented. The prevalence of RD among AFI cases was 7% (14/200); SFG and TG were identified as the cause in 4% and 3% of AFI cases, respectively. The median age of the RD cases was 22 years (range 2–65). The median duration of fever was 3 days (range 1–12). The RD cases presented with respiratory symptoms or signs (44.44%), jaundice (22.22%), abdominal pain (22.22%), diarrhea (22.22), vesicular rash (11.11%), vomiting (11.11%), loss of appetite (11.11%), headache (11.11%), leukocytosis (88.88% with mean count 22,750/mm^3^), and thrombocytopenia (33.33%). The cases were treated empirically with piperacillin-tazobactam (66.66%), clindamycin (44.44%), cefotaxime (33.33%), meropenem (33.33%), metronidazole (33.33%), doxycycline (22.22%), azithromycin (22.22%), ceftriaxone (11.11%), and amoxicillin-clavulanic acid (11.11%). The mortality among the RD cases was 11.11%. The present pilot study shows that RD is not an uncommon cause of AFI in north India. The febrile episodes are usually transient, not severe and associated with heterogenous clinical presentation without documented history of tick exposure in the hospitalized patients. The transient, non-severe, febrile illness could be due to transient rickettsemia resulting from empirical antimicrobial therapy as the rickettsial organisms are expected to be more susceptible to higher doses of β-lactam antibiotics. The study emphasizes the molecular method as a useful tool to identify rickettsial etiology in AFI.

## 1. Introduction

Rickettsial diseases (RDs) are major under-diagnosed causes of arthropod borne acute febrile illness (AFI) presenting with a range of symptoms from mild self-limiting fever to fatal sepsis. Broadly, there are two divisions of RDs: spotted fever group (SFG) caused by *Rickettsia rickettsii, Rickettsia conorii, R. sibirica, R. africae, R. parkeri, R. slovaca*, and many others and typhus group (TG) caused by *Rickettsia typhi* and *Rickettsia prowazekii.* [1]. In both, the lack of reliable serological or culture diagnostic tests is the main problem in initiating early appropriate antimicrobial therapy [2]. Therefore, diagnosis of these RDs is missed, as seen in 66.5% of scrub typhus cases [1] and in 57.9% of Mediterranean spotted fever (MSF) cases [2].

Among acute undifferentiated febrile illness (AUFI/AFI) cases from Asia, 4% of cases were caused by RD overall and among adults 12.5% of AFI cases [3]. Among the pediatric population, AFI due to RD occurred in 0.4 to 7.4% in East Africa, 2 to 17% in South Asia, 0.5 to 20.1% in SE Asia, and 7.5% in Latin America [4]. There is increasing serological evidence for RD in various parts of India [5] and molecular evidence of new rickettsial species in AFI cases [6,7]. 

In a previous study from our hospital of 51 patients who were negative for the known causes of fever, three were diagnosed to have spotted fever due to *Rickettsia conorii* [8]. In the present study, our hypothesis was that there might be a bigger burden of rickettsial etiology of AFI. Hence, in a cohort of AFI patients of undiagnosed etiology, we aimed to assess the prevalence of RD using molecular methods. 

## 2. Materials and Methods

The clearance for the study was obtained from the Institute Ethical committee (IEC number NK/447/MD/645).

### 2.1. Patients

Both inpatients and outpatients presenting to the hospital during April and May 2019 with a diagnosis of AFI were included in the study. The routine diagnostic work up for AFI was performed: blood for culture, Widal test for typhoid, scrub typhus IgM ELISA (enzyme-linked immunosorbent assay and PCR, Leptospira IgM ELISA, Brucella standard agglutination test, malaria using rapid diagnostic test kit (RDT), peripheral blood film for malaria, Dengue NS1 (nonstructural protein 1) antigen ELISA, and IgM ELISA. When all the above tests were negative in a patient’s samples, the PCR for rickettsial infections was put up. For this, the clot sample from the sample sent for serology was used.

### 2.2. DNA Extraction

To extract DNA, blood clots were broken and homogenized in a conical tube using the pipette tip and mixed with 1x lysis buffer (Qiagen QIAmp Blood mini kit) and incubated at 4 °C for 30 min. After centrifugation at 3000 rpm for 20 min, the supernatant was decanted and 1x lysis buffer was added again and centrifuged at 3000 rpm for 20 min. After the addition of 500 µL of saline EDTA (ethylenediaminetetraacetic acid), 400 µL of 0.2 M sodium acetate, 300 µL of 5% SDS (sodium dodecyl sulfate) and proteinase K, the pellets were incubated overnight at 37 °C. The DNA in this lysate was purified using phenol:chloroform:isoamyl alcohol (25:24:1) mixture, followed by precipitation with ethanol and re-suspension in 20 µL of TE (Tris EDTA) buffer. The DNA was stored in −20 °C till further molecular work up.

### 2.3. Nested PCR

A nested PCR targeting *glt*A gene of all rickettsial pathogens (TG and SFG) using the primers RpCS.877p (5’-GGGGACCTGCTCACGGCGG-3’) and RpCS.1258n (5’-ATTGCAAAAAGTACAGTGAACC-3’) in the first cycle and primers RpCS.896p (5’-GGCTAATGAAGCAGTGATAA-3’) and RpCS.1,233n (5’-GCGACGGTATACCCATAGC-3’) in nested cycle was performed in all the DNA samples extracted from clot samples. The nested PCR cycles consisted of 35 cycles of primary PCR with initial denaturation at 95 °C for 15 s, annealing at 54 °C for 15 s, extension at 72 °C for 30 s, and final extension at 72 °C for 3 min followed by 35 cycles of nested PCR with initial denaturation at 95 °C for 15 s, annealing at 54 °C for 15 s, extension at 72 °C for 30 s, and final extension at 72 °C for 3 min [6,9]. 

### 2.4. Phylogenetic Analysis

The amplicons from the nested PCR were sequenced. The phylogenetic analysis of the *Rickettsia* sequences were performed using SeqMan software v.7.0.0.0 (DNAstar, Madison, WI, USA), ClustalX 2.1 (University College Dublin, Belfield, Dublin 4, Ireland), and MEGA 7.0.14 software by the maximum likelihood analysis method. The reference *glt*A sequences of *Rickettsia* species recovered from clinical, environmental, and ticks were obtained from Pubmed Nucleotide Refsequence search (Supplementary Materials) and included to construct the phylogenetic tree using maximum likelihood analysis.

### 2.5. Clinical Details

The clinical details were retrieved retrospectively for the samples which were positive for rickettsial pathogens. All demographic details, clinical features including rashes and eschar, exposure to ticks and animals, radiological and hematological investigations, treatment histories, and the outcome of all rickettsial PCR positive cases were searched and documented. The analysis was done using Microsoft Excel.

## 3. Results

The sequences were submitted to GenBank (accession numbers MN497608−MN497621) and they were 99–100% homologous with more than one rickettsial species. We found they fell into two groups: SFG rickettsial species and TG rickettsial species; the details shown in Supplementary Materials. Among 200 consecutive samples, 14 (7%) were positive for *glt*A nested PCR. Among the 14 sequences, eight were suggestive of SFG *Rickettsia* species and six of TG *Rickettsia*.

Among 14 patients, 11 (78.6%) were adults and 8 (57.1%) were female. The median age of the RD cases was 22 years (range 2−65). Eleven patients were inpatients and medical records were available to us in nine of these patients only (Table 1 and Table 2). The median duration of fever was three days (range 1–12). There was no mention of any eschar or a history of tick bite in any patient’s records. The patients had been treated empirically with piperacillin-tazobactam (66.66%), clindamycin (44.44%), cefotaxime (33.33%), meropenem (33.33%), metronidazole (33.33%), doxycycline (22.22%), azithromycin (22.22%), ceftriaxone (11.11%), and amoxicillin-clavulanic acid (11.11%). There was poor lethal outcome in a single patient in our cohort who was started with doxycycline on the day of admission and the drug was continued throughout his hospital stay and the patient succumbed to death on the fourth day of admission. 

**Figure 1:**
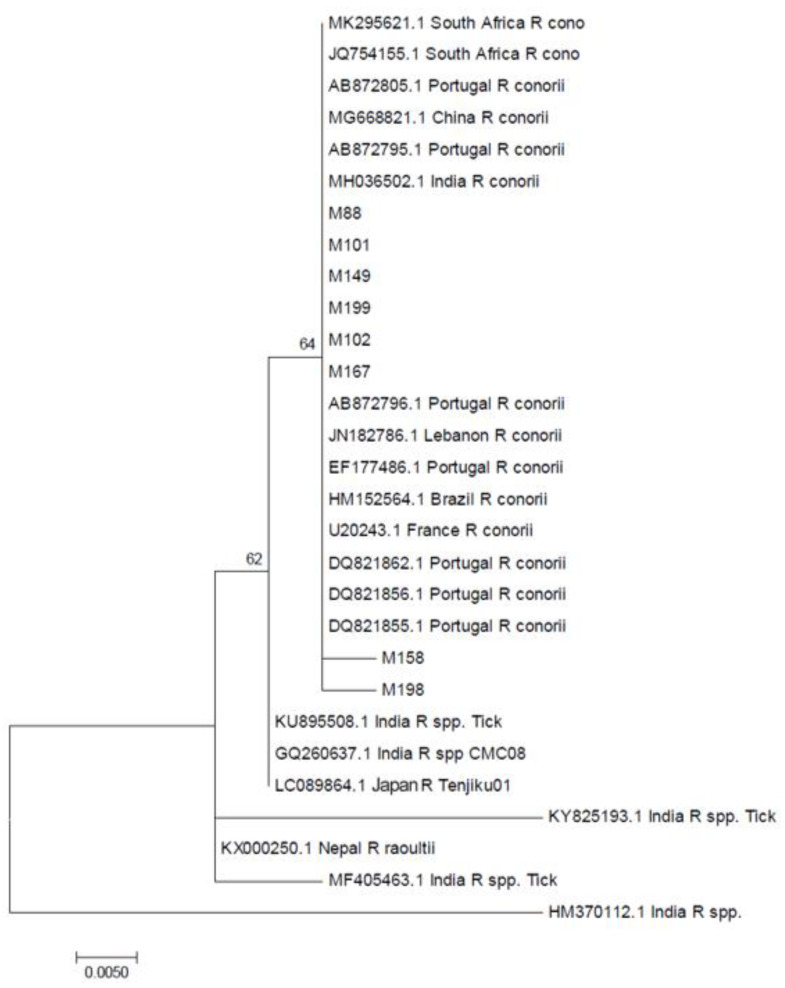
A phylogenetic tree constructed for spotted fever group (SFG) *Rickettsia* species sequences from our study based on *gltA* gene sequences using MEGA7 software.

**Figure 2:**
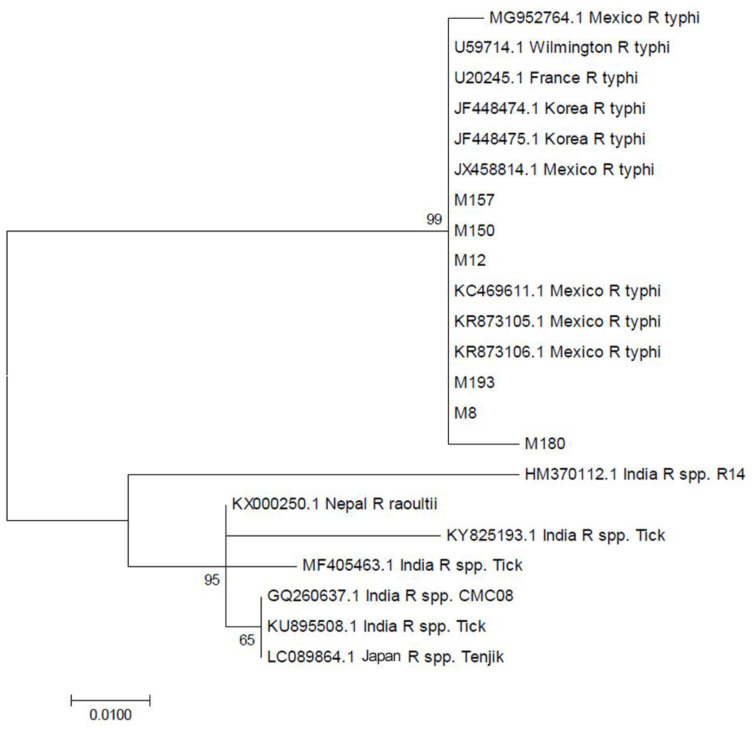
A phylogenetic tree constructed for typhus group (TG) *Rickettsia* species sequences from our study based on *gltA* gene sequences using MEGA7 software.

**Figure 3:**
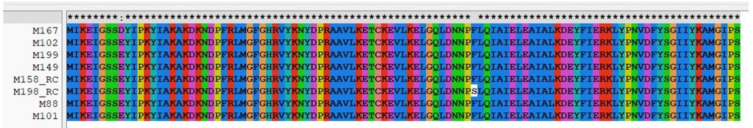
The amino acid change in the citrate synthase gene (*gltA*) product of sequence of SFG *Rickettsia* species M198.

The phylogenetic analysis is shown in Figure 1 and Figure 2. The SFG *Rickettsial* species sequences from our study (M88, M101, M102, M149, M167, M199) are very similar to the available *gltA* reference and other partial sequences of *R. conorii* from the GenBank, except two (M158 and M198; Figure 3). The patients of these sequences were treated with clindamycin. Our sequences were similar to SFG *Rickettsial* species *gltA* sequence (MH036502.1) previously submitted from India, which was from a hospital-based surveillance in North India [10]. A sequence from a Japanese traveler who had returned from India (Rickettsia species Tenjiku01) [7], ticks, and environment *gltA* partial sequences (KU895508.1, GQ260637.1, KY825193.1, KX000250.1, MF405463.1, HM370112.1) (Supplementary Materials) were used as outgroups. 

There was an amino acid substitution at position 64 from phenylalanine to serine (F64S) noted in M198 when compared with other SFG *Rickettsia* species sequences from our study (Figure 3). The change in amino acids could possibly be explained by selection of the mutant strain due to selective pressure of treatment with clindamycin, which is a protein synthesis inhibitor and also might inhibit the mitochondrial protein synthesis [11].

In M180, there was a one nucleotide difference but there was no change in the corresponding amino acid sequence.

All TG *Rickettsial* species sequences from our study (M8, M12, M150, M157, M193) were also found to be very similar to the available *gltA* reference and other partial sequences of *R. typhii* from the GenBank. This patient presented with a vesicular rash. No environmental or tick associated *R. conorii* or *R. typhi gltA* sequences were available from the GenBank to use as an outgroup in the analysis. 

## 4. Discussion

Rickettsial infections are hugely underdiagnosed especially in India. The reported seropositivity in clinically suspected infections is up to 33% [12]. Many states in India have serological evidence of the presence of these infections [12]. In a study on AFI patients in 2015, the IgM antibody against SFG and TG was 13.6% and 7.1%, respectively [13]. The present study used PCR to detect a prevalence of 7% in AFI (spotted fever group was 4% and typhus group was 3%). 

Due to the retrospective nature of clinical and epidemiological data collection in the present study, it is possible that many details are missing. For example, occupation, history of travel, tick exposure history, etc., were mentioned in the records. In addition, rash and eschar might not be looked for, especially in non-exposed body parts as the diagnosis was made much later. Additionally, in dark-skinned individuals, a transient rash might be easily missed. Therefore, discussion about these points can only be based on whatever details could be collected. 

A good proportion of our patients presented in the peripartum period. There are fewer reports of *Rickettsia* infections reported during pregnancy compared to scrub typhus. Mathai et al. reported five patients with scrub typhus during pregnancy from India [14,15]. Mane et al. detected higher SFG IgM positivity in females and higher IgM seropositivity for TG rickettsiae in housewives [13]. Clinically, McGready et al. reported that scrub typhus and murine typhus are indistinguishable with a poor neonatal outcome in 36% of patients [16].

There was no record of any eschar in our patients. As mentioned before, it could be because it was specifically not looked for. Furthermore, not all rickettsioses present with an eschar [2]. There is a much higher prevalence of eschars in MSF caused by *R. conorii conorii*, Malish strain compared to Israeli spotted fever caused by *R. conorii subsp. Israelensis.* Although mostly benign, MSF has a mortality rate of 2.5%, and the prevalence of severe forms of the disease is increasing [17]. The mortality rate in our study was 11.11% which was relatively higher. 

Only three of the nine patients received anti-rickettsial drugs. Notwithstanding this, the majority of them improved. This could point to a self-limiting course of the disease. The febrile episodes are usually transient, not severe, and sometimes without typical presentation (rash, eschar) [18,19]. As with other species of rickettsiae, penicillins are ineffective [17]. Classically, beta-lactams and aminoglycosides are not effective against rickettsia. However, Wisseman et al. showed that both penicillin G and gentamicin at certain concentrations have significant inhibitory actions on *R. prowazekii* plaque formation, and that penicillin G induces the formation of spheroplasts [20,21]. All our patients had penicillin or cephalosporin administered and showed improvement without an anti-rickettsial drug. It is not clear if these antibiotics may have aided the recovery. 

The study emphasizes that the molecular method is a useful tool to identify the rickettsial cause of AUFI. In our study, we targeted the *glt*A gene in the molecular screening of rickettsial infections which can be helpful in genus level identification with the possibility to differentiate into groups (SFG, TG) but not able to differentiate different species of Rickettsiae. Other genes/loci such as *omp*A, *omp*B, rrs, and *htr*A are more useful after the screening with *glt*A gene for accurate species level identification [22]. 

We found dozens of rickettsial species and strains with 99–100% homology when used with *glt*A as a screening tool and it warrants the use of other targets for species level identification of Rickettsiae. A limitation of our study includes not using other targets to confirm the species in our cohort and there are very few molecular studies from India, and we have attempted to look for Rickettsial infections in our center. This is an initial pilot study aimed to highlight the magnitude of this infection in patients with acute undifferentiated febrile illness in India. These initial findings will be helpful to plan a systematic study using multiple gene targets in the near future. Prospective studies are required to elucidate the clinical features, risk factors, and molecular epidemiology of rickettsioses in our region. 

## 5. Conclusions

Both spotted fever rickettsioses and murine typhus is not uncommon in India. Clinicians working in India, or treating travelers returning from India, should have a low threshold of suspicion for rickettsial disease. Due to the low sensitivity of serological tests, molecular tests should be conducted to diagnose these infections.

## Figures and Tables

**Table 1:** Clinical features, investigations performed, and final outcome of patients infected with spotted fever group *Rickettsia* species.

Case	Age, Gender	ID	Diagnosis	Clinical Feature (Number of Days)	Hemogram/LFT */RFT */ Coagulogram Abnormality/Sepsis Markers	Treatment	Outcome
**1**	22, F	M88	Sickle cell anemia, beta thalassemia, asplenia	Fever (3), myalgia (1)	Leukocytosis, anemia, elevated alkaline phosphate, procalcitonin 1.8 ng/mL	Cefotaxime, metronidazole, clindamycin, piperacillin-tazobactam	Deteriorated, discharged on request
**2**	31, F	M149	Fever in pregnancy	Fever (4), abdominal pain	Leukocytosis, anemia	Cefotaxime, metronidazole, clindamycin, piperacillin-tazobactam	Discharged
**3**	28, F	M158	Fever in pregnancy, pneumonia	Fever (1), cough (4), hemoptysis (1), shortness of breath (4), bilateral middle and lower zone pulmonary opacities	Leukocytosis, anemia	Amoxicillin-clavulanic acid, piperacillin-tazobactam, clindamycin	Discharged
**4**	22, F	M167	Acute severe hepatitis, severe sepsis, MODS *	fever (5), jaundice (5), epigastric pain (5), shortness of breath (1), loss of appetite (30), axillary lymphadenopathy	Leukocytosis, anemia, elevated liver function parameter	Doxycycline (4), meropenem, teicoplanin	Death
**5**	32, F	M198	Fever in pregnancy, PPH *, shock	Fever on and off during admission, hepatomegaly, cholelithiasis	Leukocytosis, hyperbilirubinemia, thrombocytopenia	Piperacillin-tazobactam, clindamycin, meropenem, vancomycin	Discharge

* MODS: multi organ dysfunction syndrome, PPH: postpartum hemorrhage, LFT: liver function test, RFT: renal function test.

**Table 2:** Clinical features, investigations performed, and final outcome of patients infected with typhus group *Rickettsia* species.

Case	Age, Gender	ID	Diagnosis	Clinical Feature (Number of Days)	Hemogram/LFT/RFT/Coagulogrm Abnormality/Sepsis Markers	Treatment	Outcome
**1**	65, F	M8	Adrenal malignancy, sepsis-induced ARDS *	Fever (3), diarrhea (4), lower abdominal pain (30), shortness of breath (1), bilateral basal lung infiltrates	Leukocytosis, anemia, elevated procalcitonin	Cefotaxime, amikacin, meropenem, metronidazole	Discharged
**2**	22, F	M193	23 weeks pregnant, hemolytic disease of newborn	Fever (2), cough (1), headache (5)	Leukocytosis, anemia, procalcitonin 1.38 ng/mL	Ceftriaxone, azithromycin	Discharged
**3**	40, M	M180	CKD *, renal transplan, On Chemotherapy and steroids, herpes zoster	Fever (4), erythematous vesicular rash on right hemithorax, vomiting (5), diarrhea (5),	Leukopenia, anemia, increased urea and creatinine, thrombocytopenia	Piperacillin-tazobactam, acyclovir	Discharged
**4**	26, M	M150	AFI *, pneumonia	Fever (12), rash, diarrhea, abdominal pain, mild splenomegaly	Leukocytosis, anemia, thrombocytopenia, elevated creatinine and alkaline phosphate	Doxycycline (5), azithromycin (5), piperacillin-tazobactam, vancomycin	Discharged

* ARDS: acute respiratory distress syndrome, CKD: chronic kidney disease, AFI: acute febrile illness.

## Supplementary Materials

The following are available online at https://www.mdpi.com/2414-6366/5/2/59/s1.
